# Supplementary material for: NU7441, a selective inhibitor of DNA-PKcs, alleviates intracerebral hemorrhage injury with suppression of ferroptosis in brain
Source: PeerJ. 2024 Nov 19;12:e18489. doi: 10.7717/peerj.18489 (PMC11583913; doi:10.7717/peerj.18489)
Supplement: Supplemental Information 3 [file peerj-12-18489-s003.pdf]

FIg1C

(1)

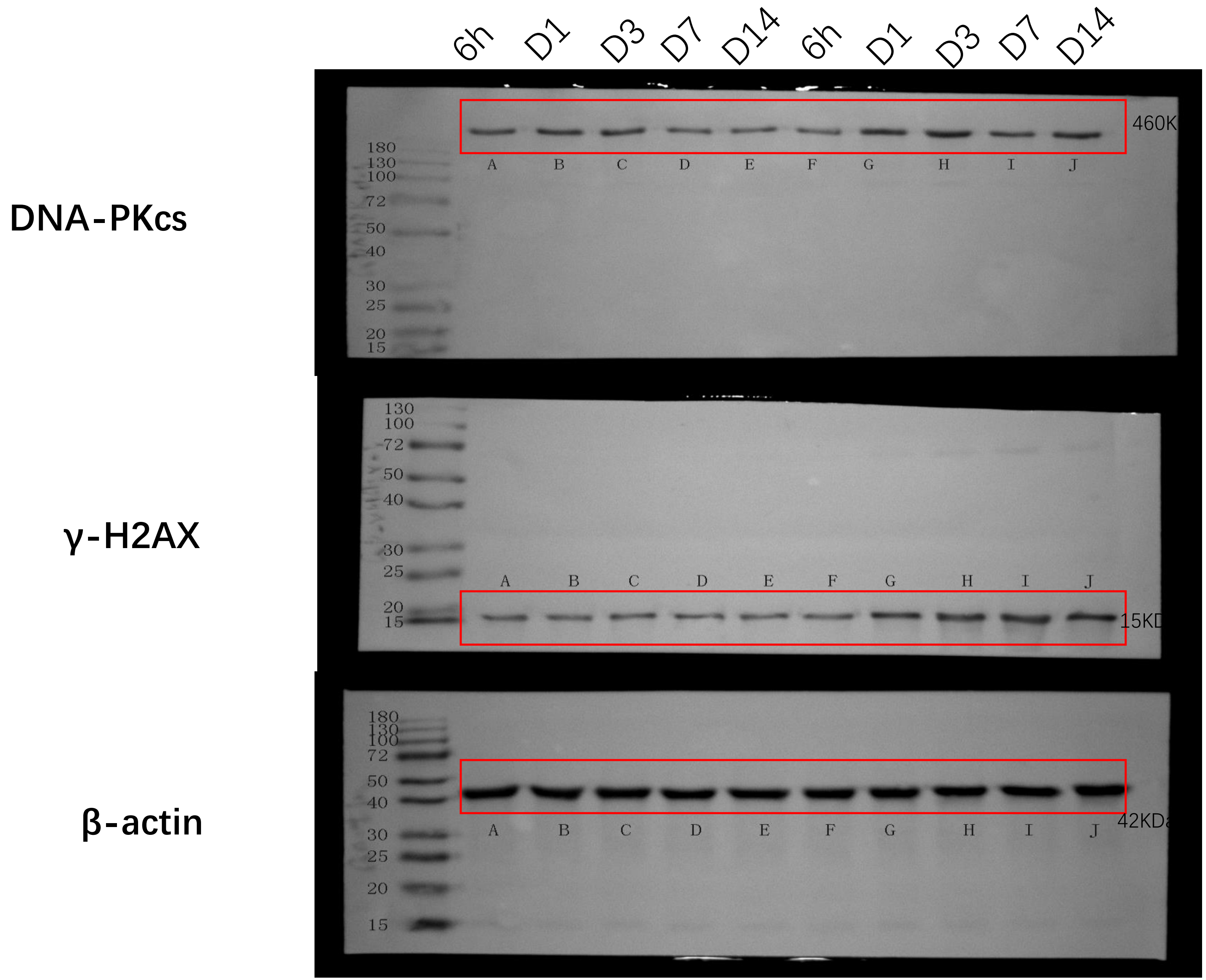
6h
D1
D3
D7
D14
6h
D1
D3
D7
D14
DNA-PKcs
460K
γ-H2AX
15KD
β-actin
42KDa
180
130
100
72
50
40
30
25
20
15
A
B
C
D
E
F
G
H
I
J

FIg1C

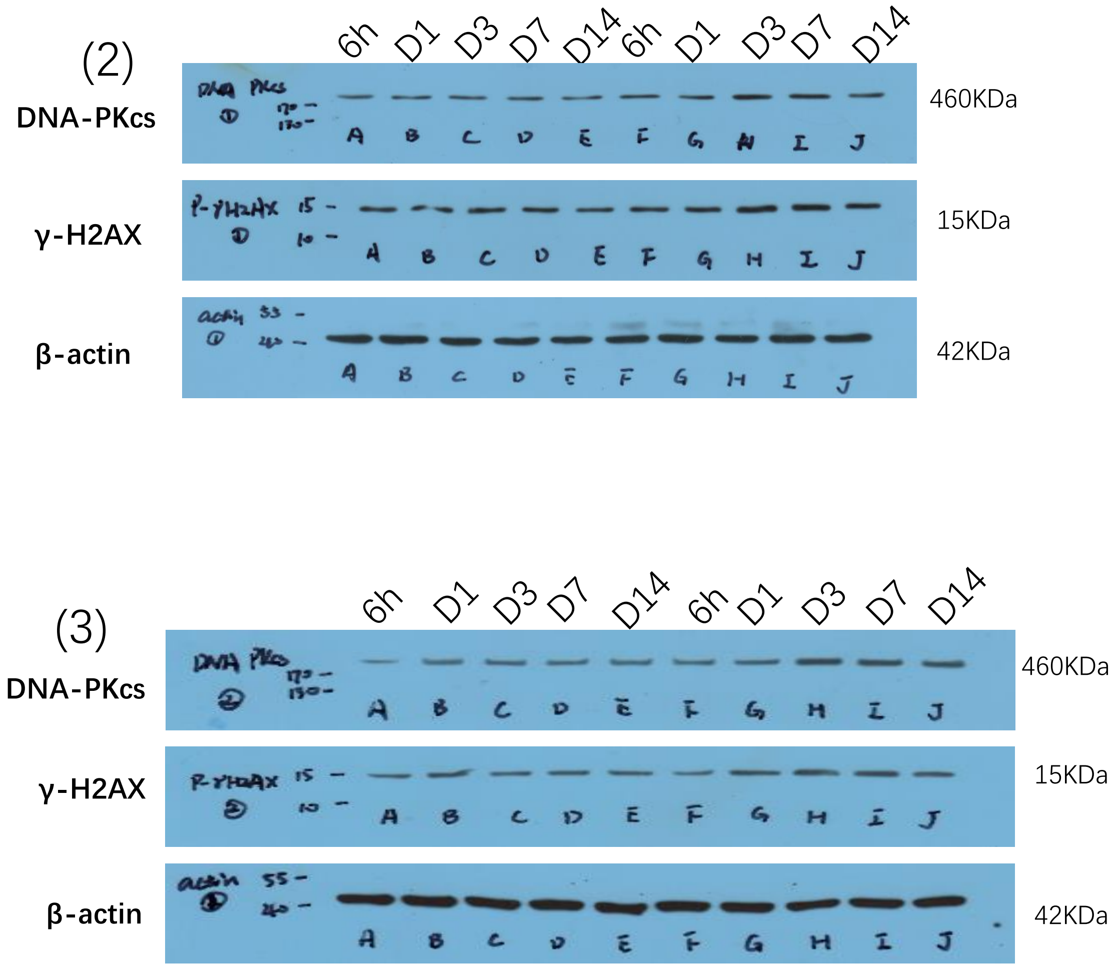
(2)
6h D1 D3 D7 D14 6h D1 D3 D7 D14
DNA-PKcs
460KDa
γ-H2AX
15KDa
β-actin
42KDa
(3)
6h D1 D3 D7 D14 6h D1 D3 D7 D14
DNA-PKcs
460KDa
γ-H2AX
15KDa
β-actin
42KDa

FIg1E

(1)

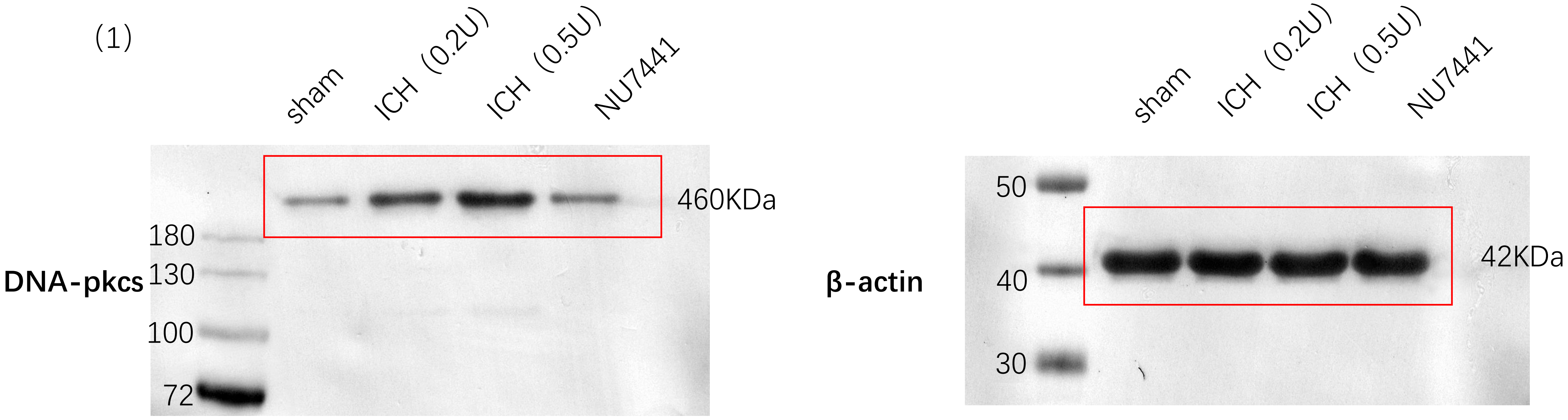

sham
ICH（0.2U）
ICH（0.5U）
NU7441
460KDa
180
130
100
72
DNA-pkcs
β-actin
50
40
30
42KDa

FIg1E

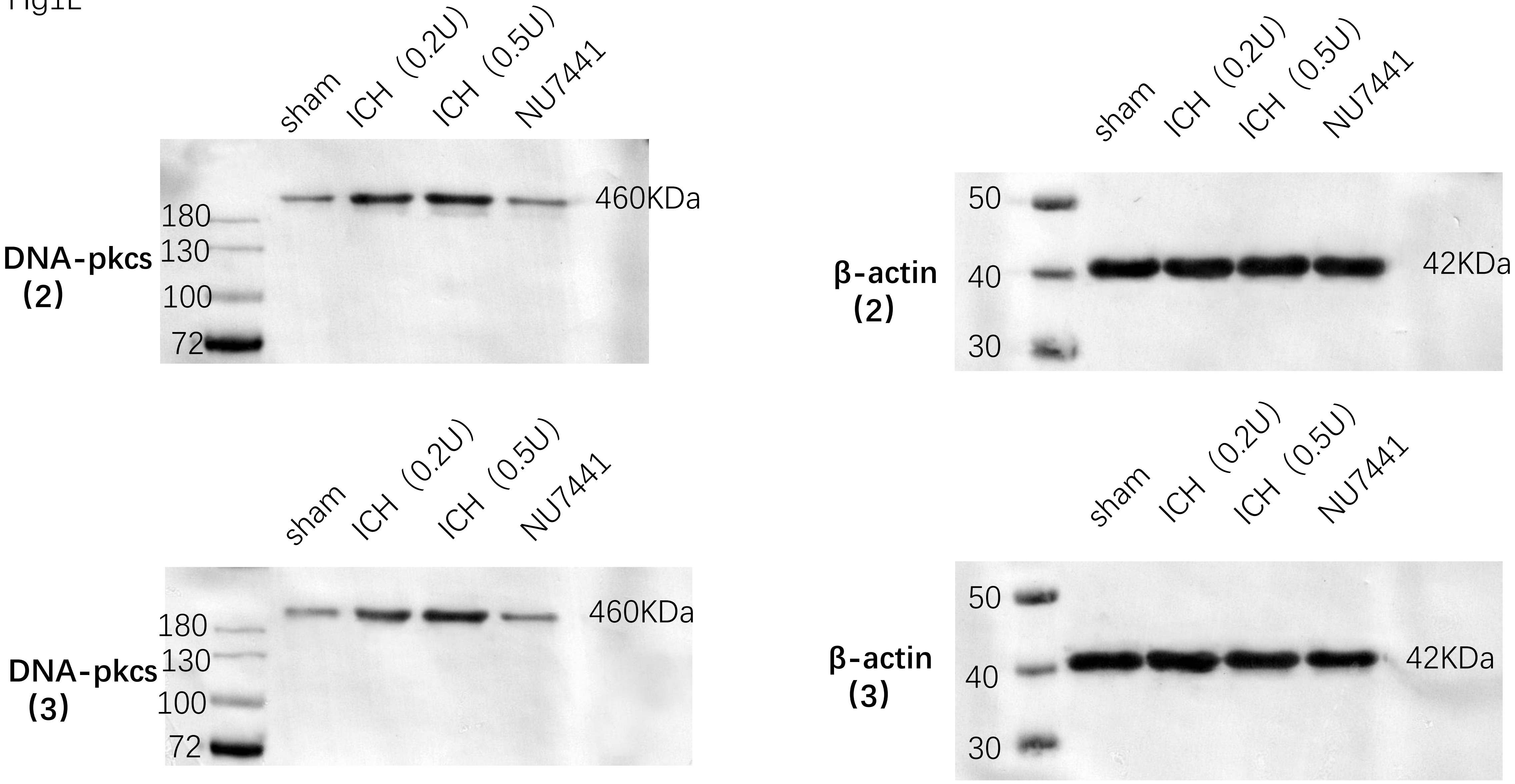
sham
ICH（0.2U）
ICH（0.5U）
NU7441
DNA-pkcs
(2)
180
130
100
72
460KDa
β-actin
(2)
50
40
30
42KDa
sham
ICH（0.2U）
ICH（0.5U）
NU7441
DNA-pkcs
(3)
180
130
100
72
460KDa
β-actin
(3)
50
40
30
42KDa

FIg2A

(1)

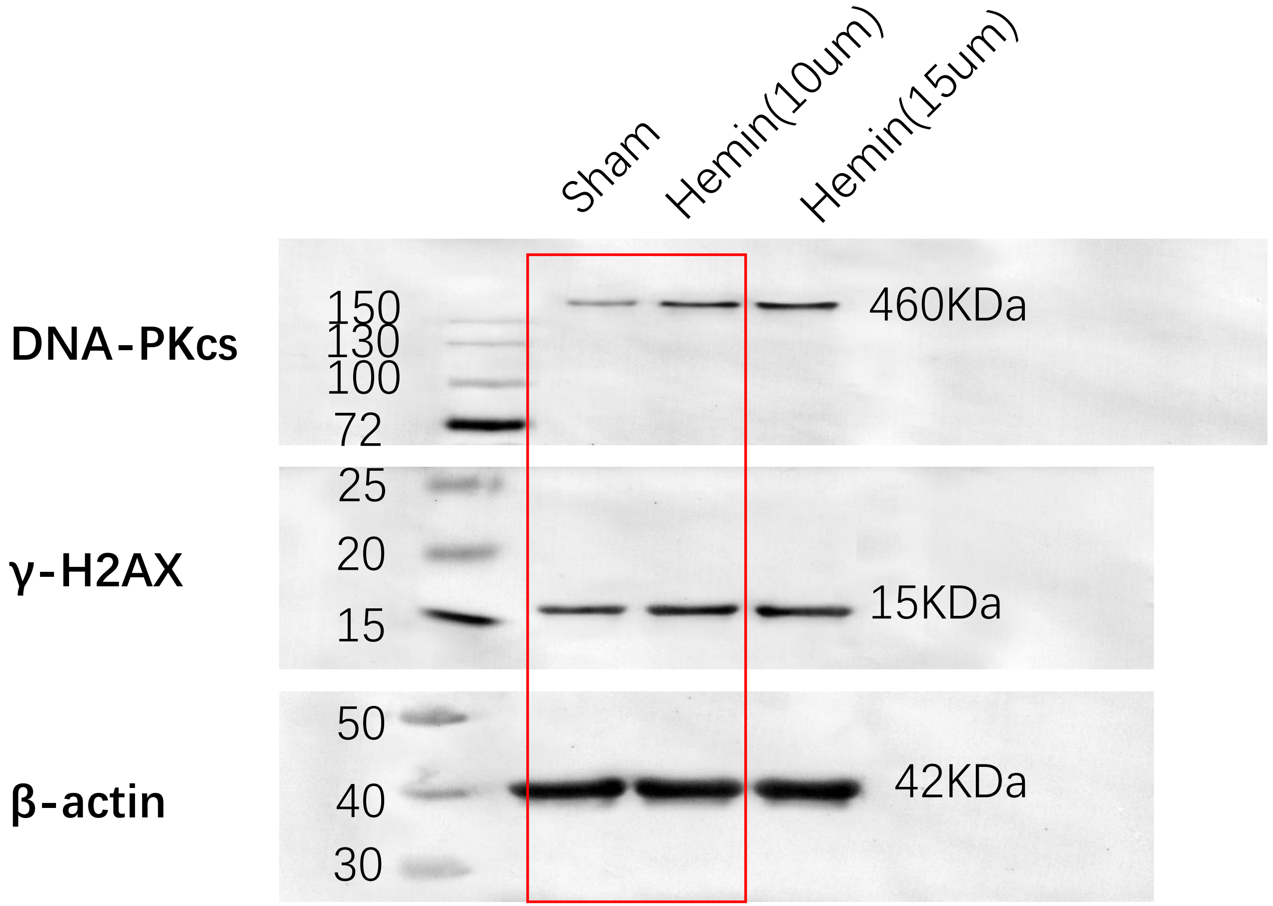
Sham
Hemin(10um)
Hemin(15um)
DNA-PKcs
150
130
100
72
460KDa
γ-H2AX
25
20
15
15KDa
β-actin
50
40
30
42KDa

FIg2A

(2)

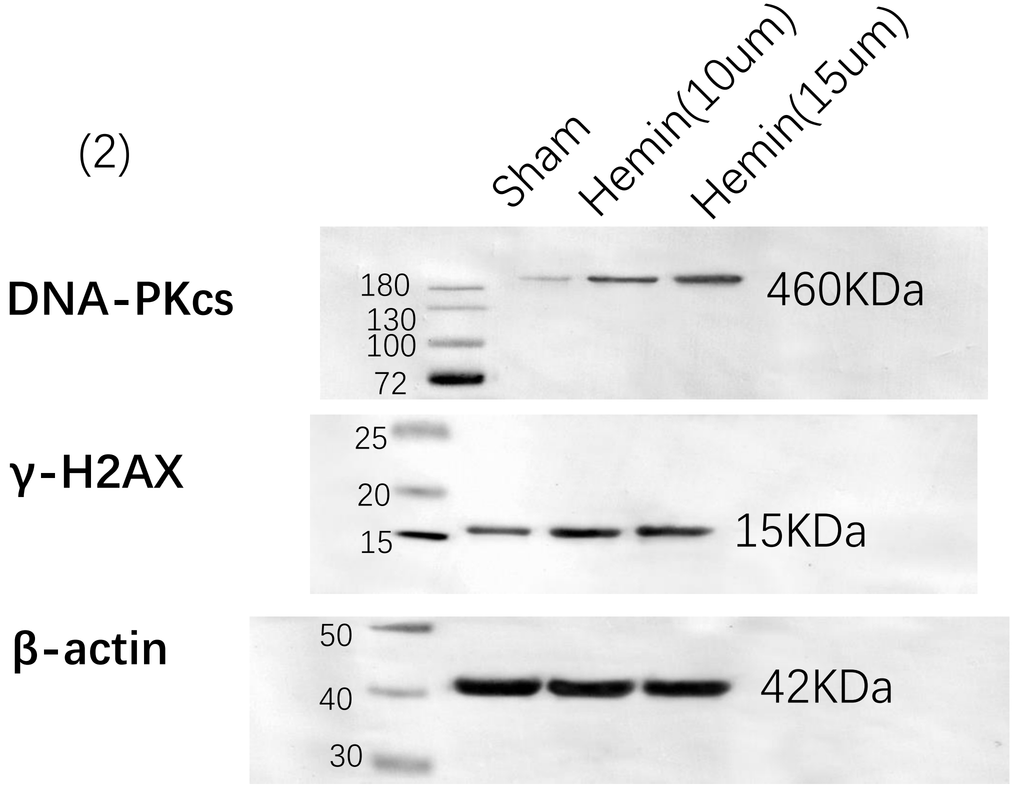
Sham
Hemin(10um)
Hemin(15um)
DNA-PKcs
180
130
100
72
460KDa
γ-H2AX
25
20
15
15KDa
β-actin
50
40
30
42KDa

(3)

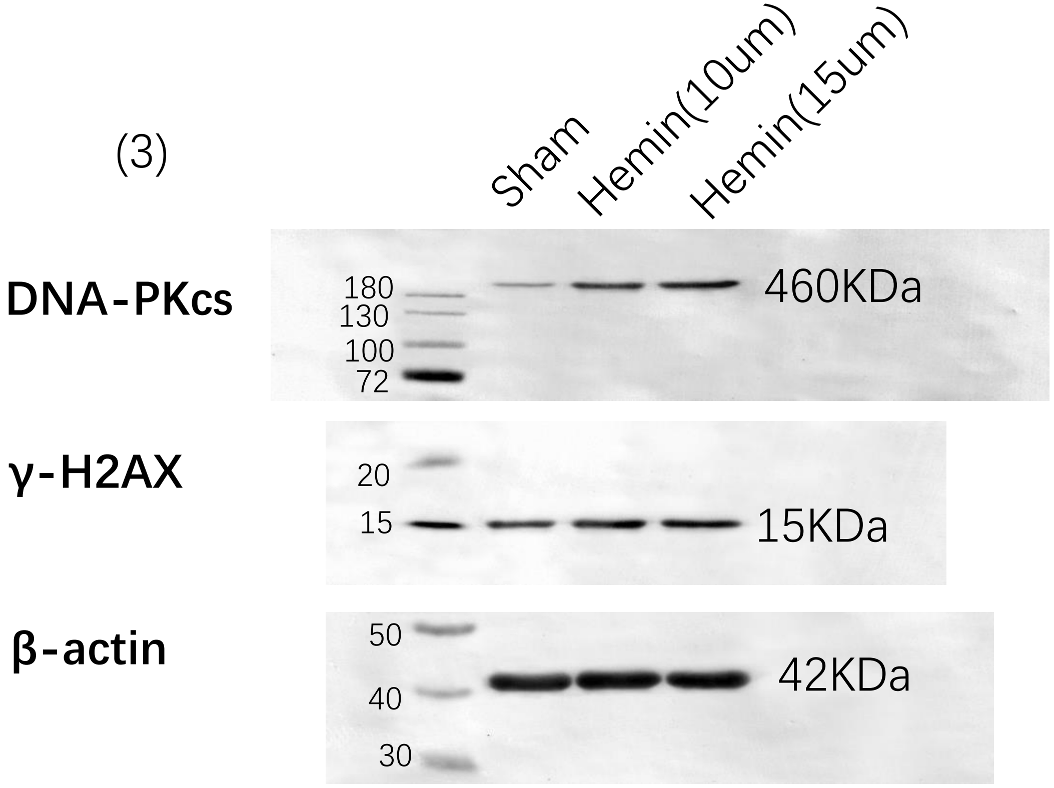
Sham
Hemin(10um)
Hemin(15um)
DNA-PKcs
180
130
100
72
460KDa
γ-H2AX
20
15
15KDa
β-actin
50
40
30
42KDa

FIg7A

(1)

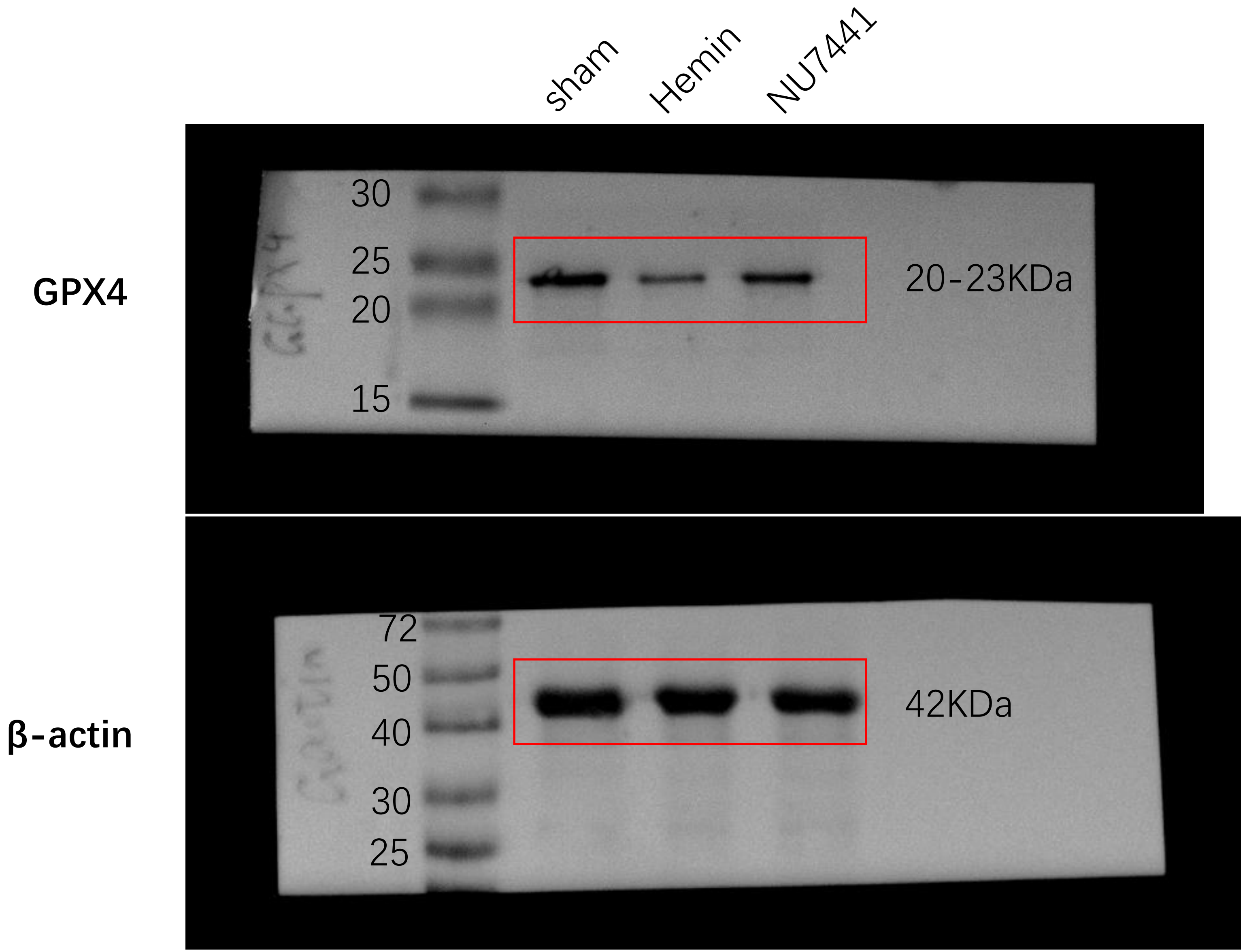
sham
Hemin
NU7441
GPX4
30
25
20
15
20-23KDa
β-actin
72
50
40
30
25
42KDa

FIg7A

(2)

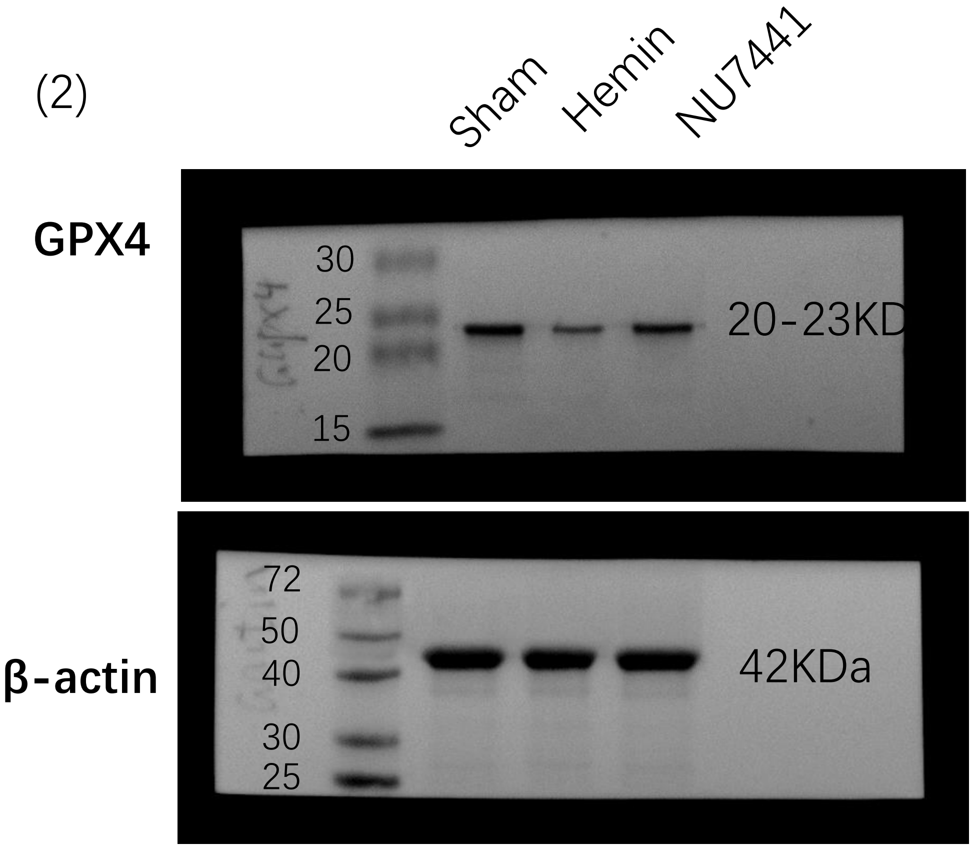
Sham
Hemin
NU7441
GPX4
30
25
20
15
20-23KD
β-actin
72
50
40
30
25
42KDa

(3)

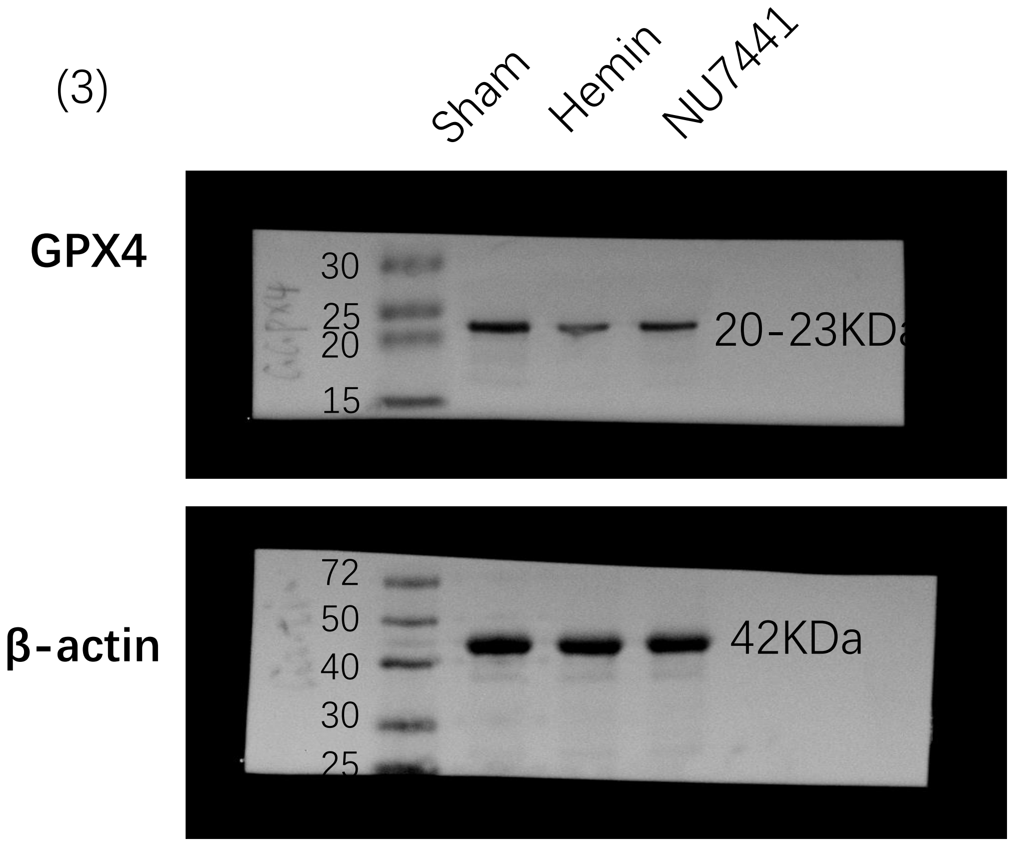
Sham
Hemin
NU7441
GPX4
30
25
20
15
20-23KDa
β-actin
72
50
40
30
25
42KDa

FIg8A

(1)

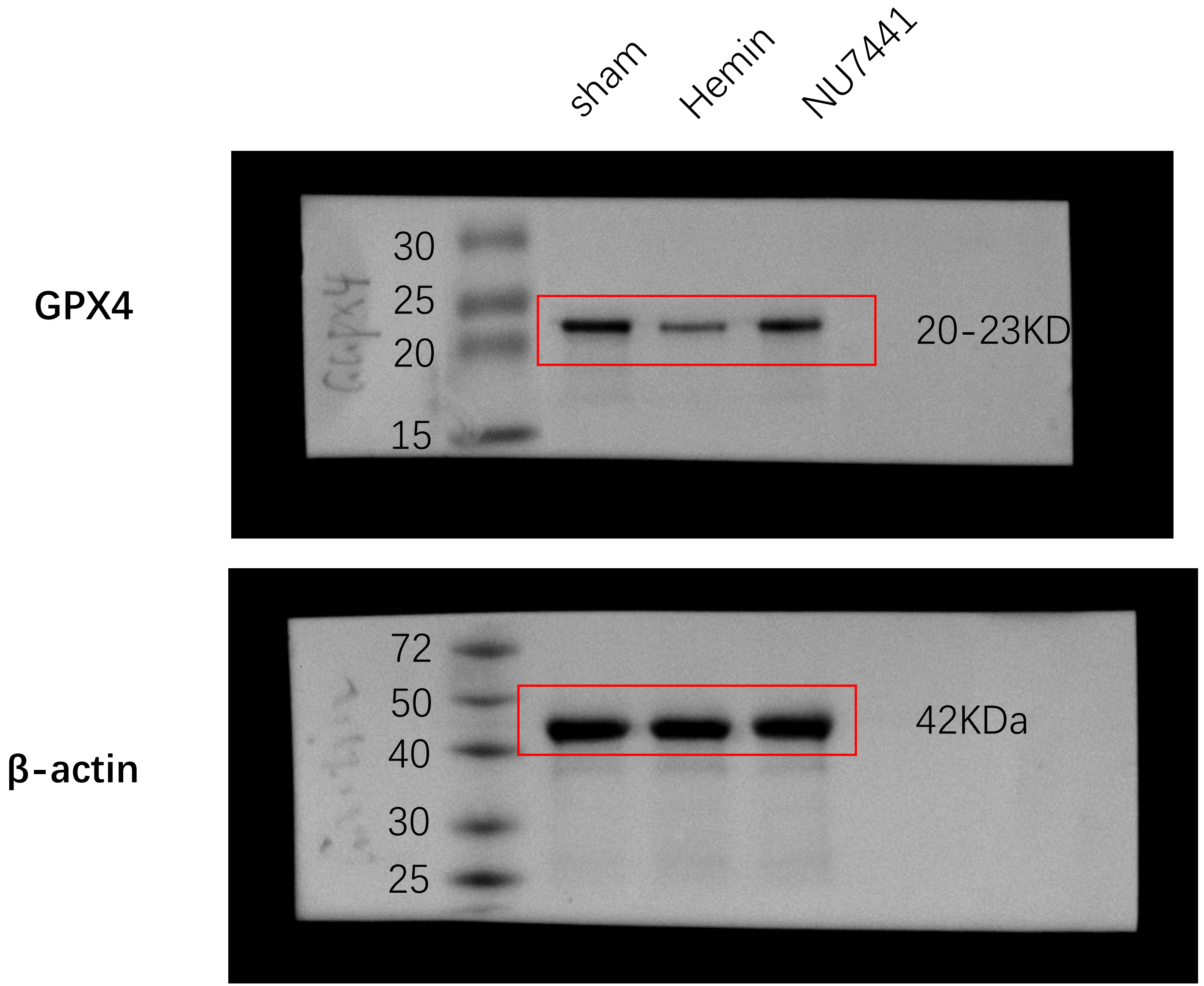
sham
Hemin
NU7441
GPX4
30
25
20
15
20-23KD
β-actin
72
50
40
30
25
42KDa

FIg8A

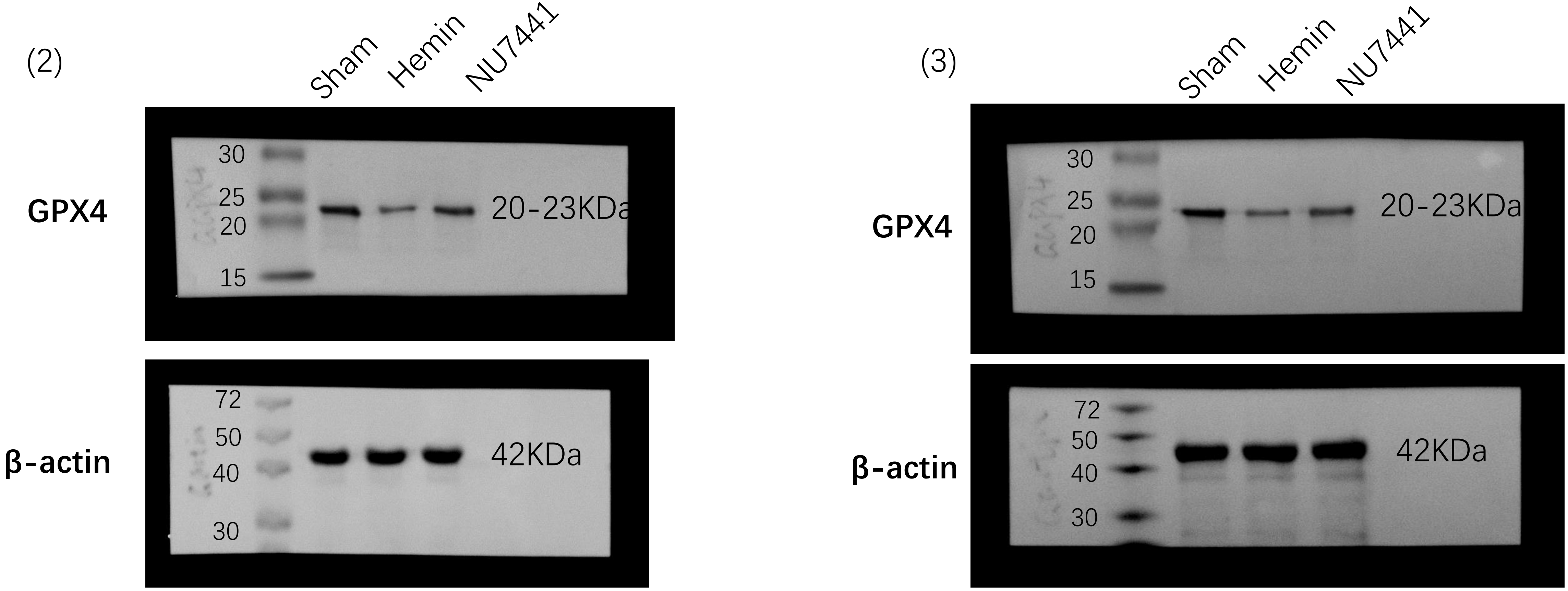
(2)
Sham
Hemin
NU7441
GPX4
30
25
20
15
20-23KDa
β-actin
72
50
40
30
42KDa
(3)
Sham
Hemin
NU7441
GPX4
30
25
20
15
20-23KDa
β-actin
72
50
40
30
42KDa
