# Supplementary figures and images for: NU7441, a selective inhibitor of DNA-PKcs, alleviates intracerebral hemorrhage injury with suppression of ferroptosis in brain
Source: PeerJ. 2024 Nov 19;12:e18489. doi: 10.7717/peerj.18489 (PMC11583913; doi:10.7717/peerj.18489)

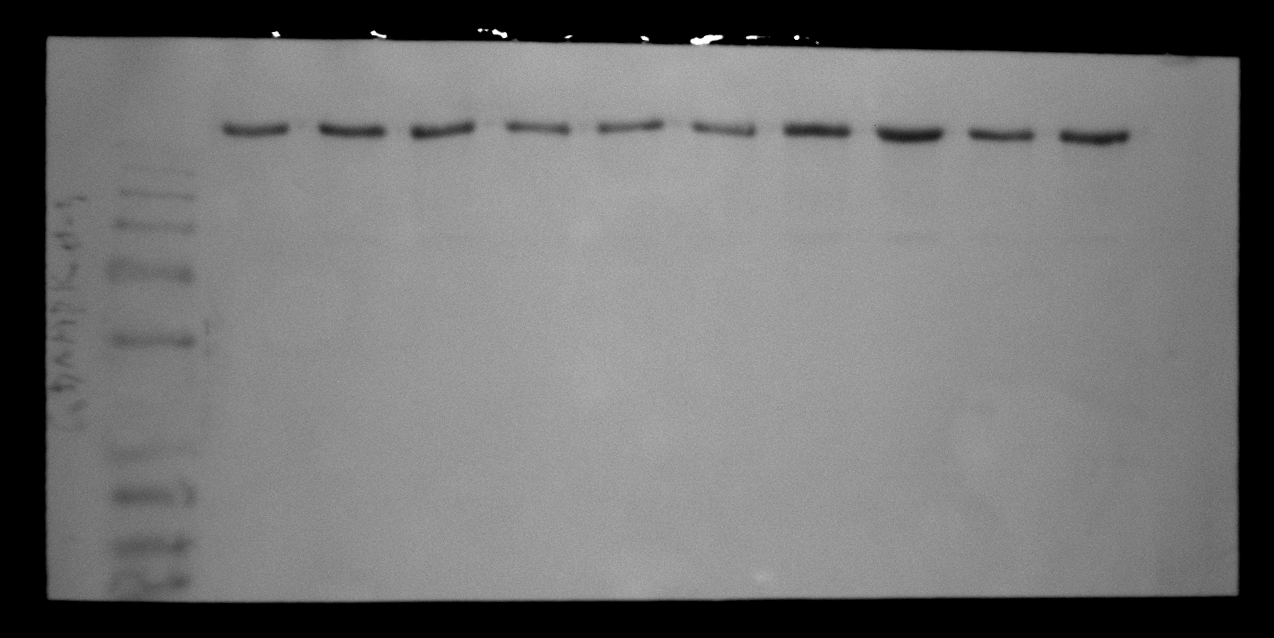

Supplement: Supplemental Information 5 [file peerj-12-18489-s005.zip › Uncropped Blots/figure1C/DNA PKcs.tif]

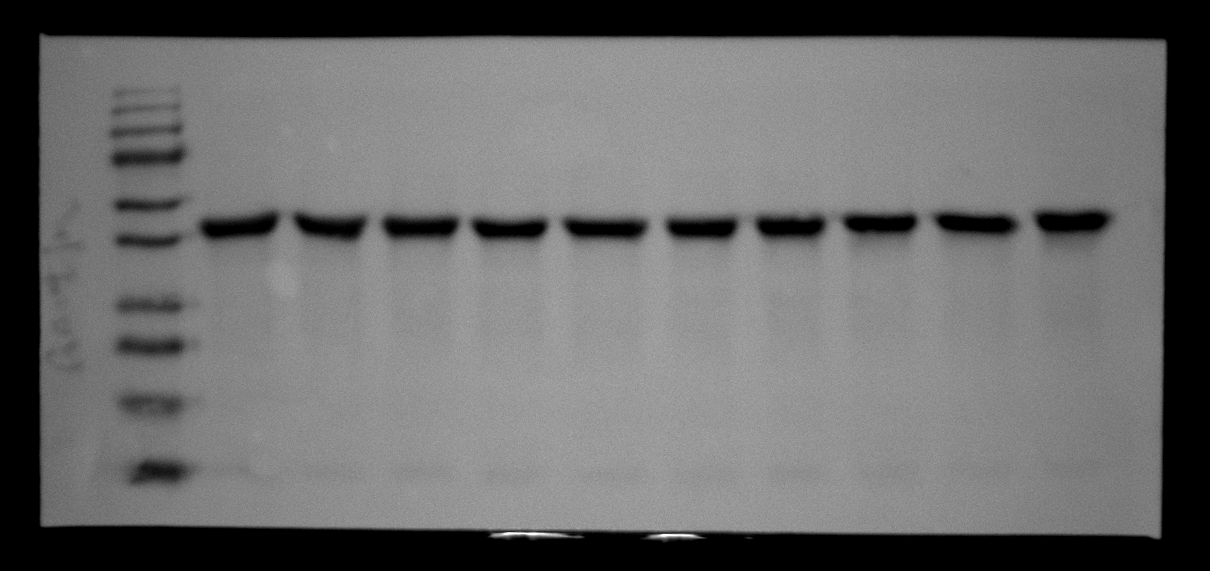

Supplement: Supplemental Information 5 [file peerj-12-18489-s005.zip › Uncropped Blots/figure1C/actin.tif]

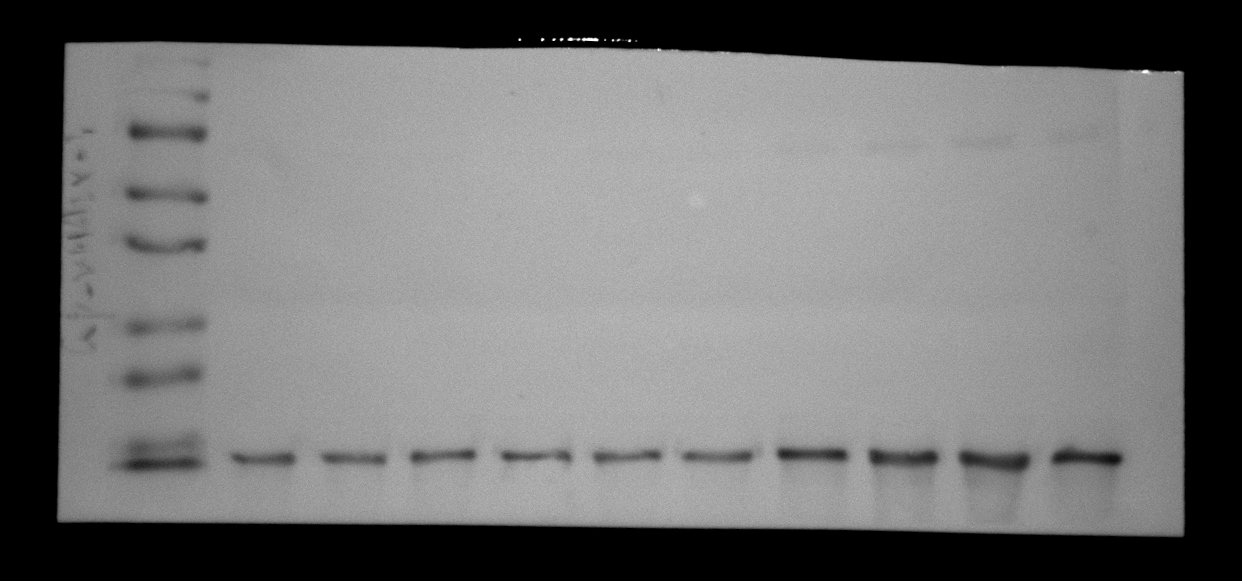

Supplement: Supplemental Information 5 [file peerj-12-18489-s005.zip › Uncropped Blots/figure1C/p-γH2AX.tif]

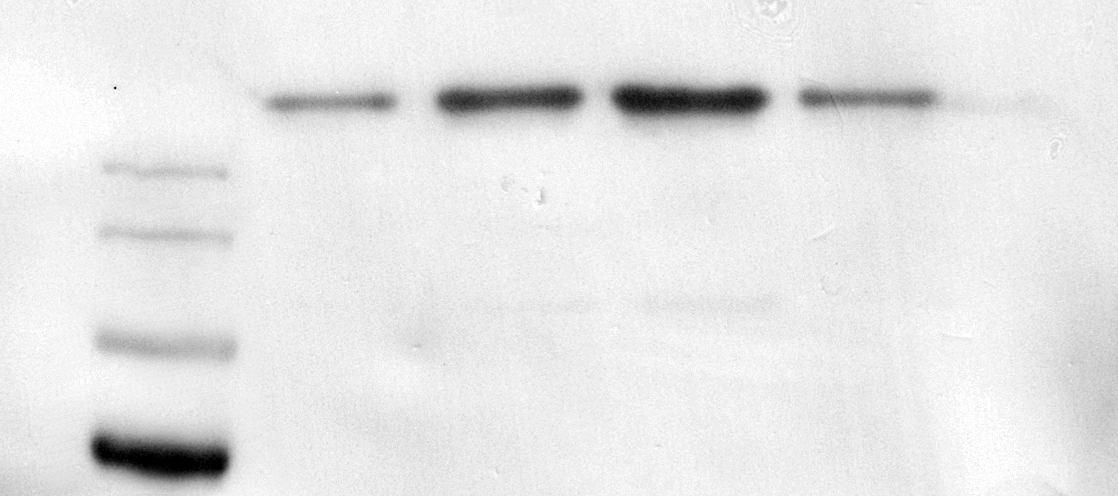

Supplement: Supplemental Information 5 [file peerj-12-18489-s005.zip › Uncropped Blots/figure1E/DNAPKcs.tif]

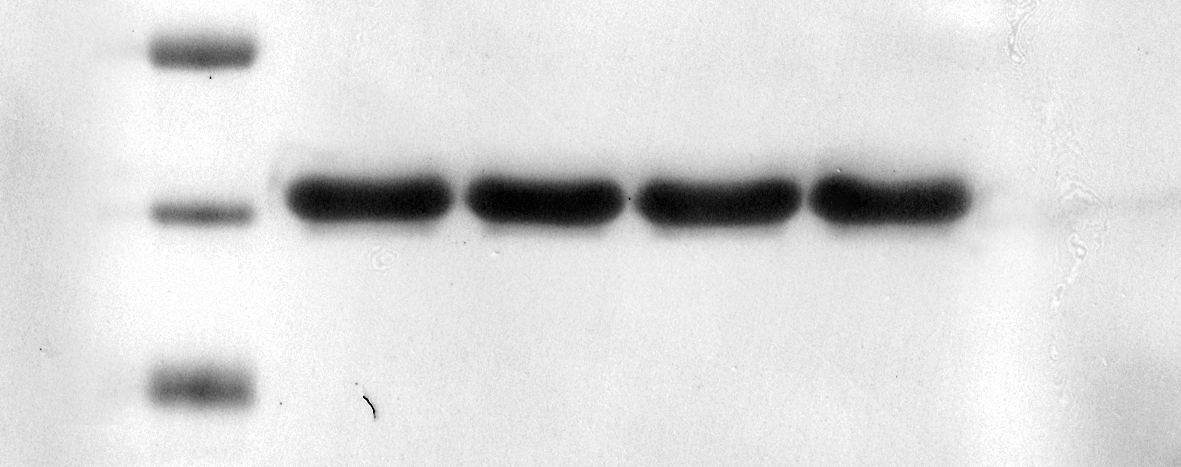

Supplement: Supplemental Information 5 [file peerj-12-18489-s005.zip › Uncropped Blots/figure1E/actin.tif]

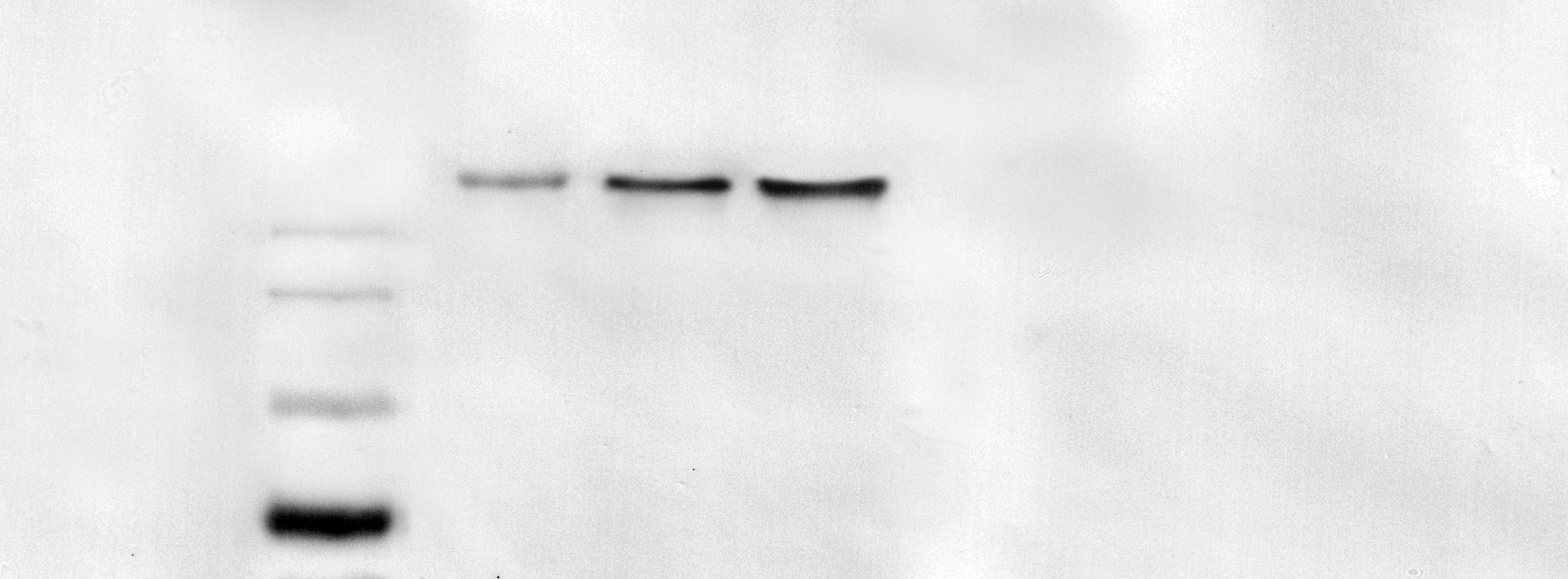

Supplement: Supplemental Information 5 [file peerj-12-18489-s005.zip › Uncropped Blots/figure2A/DNAPK-1.tif]

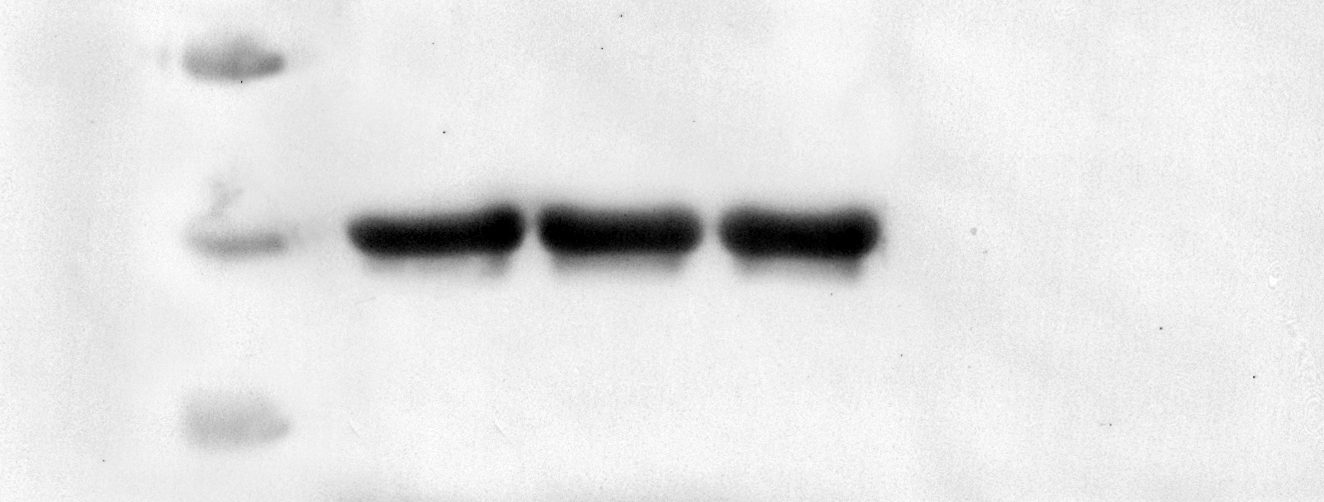

Supplement: Supplemental Information 5 [file peerj-12-18489-s005.zip › Uncropped Blots/figure2A/actin-1.tif]

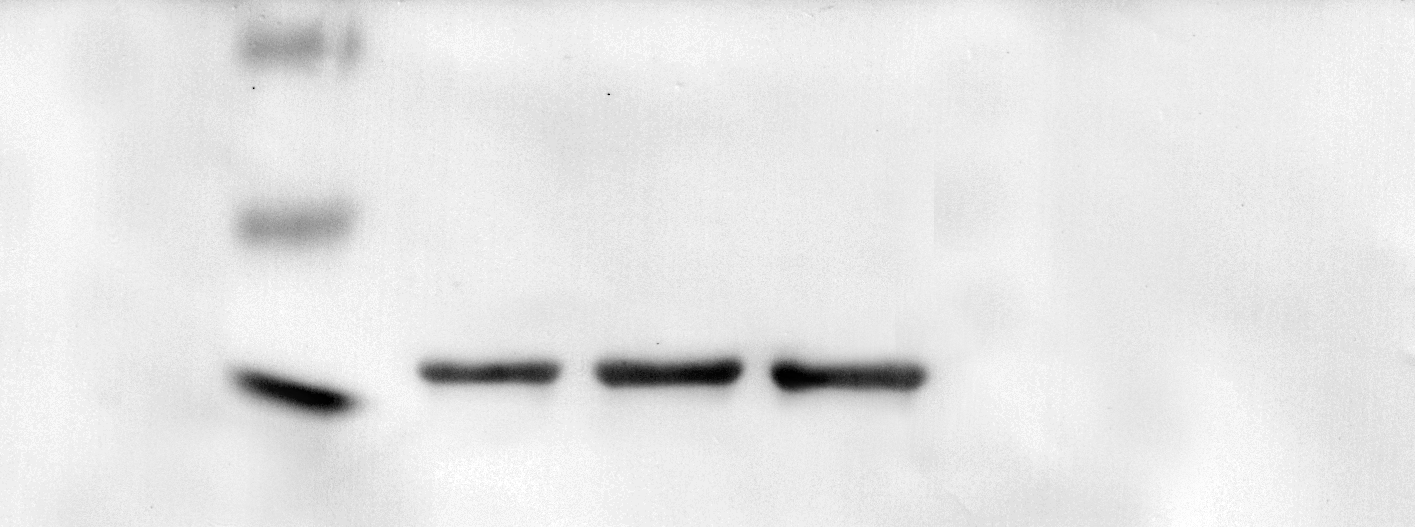

Supplement: Supplemental Information 5 [file peerj-12-18489-s005.zip › Uncropped Blots/figure2A/γ-H2AX-1.tif]

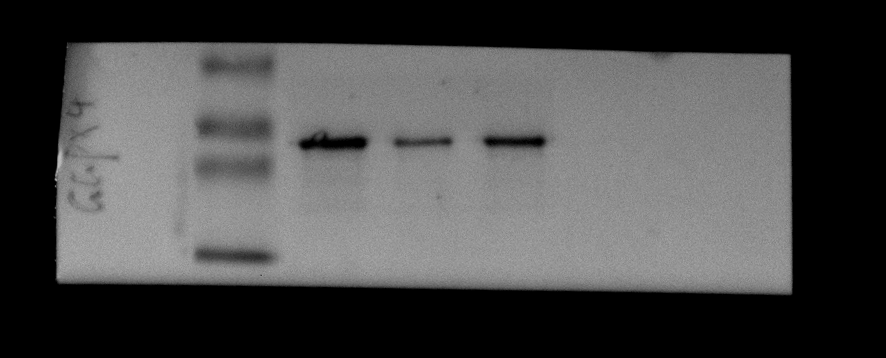

Supplement: Supplemental Information 5 [file peerj-12-18489-s005.zip › Uncropped Blots/figure7A/GPX4-1.tif]

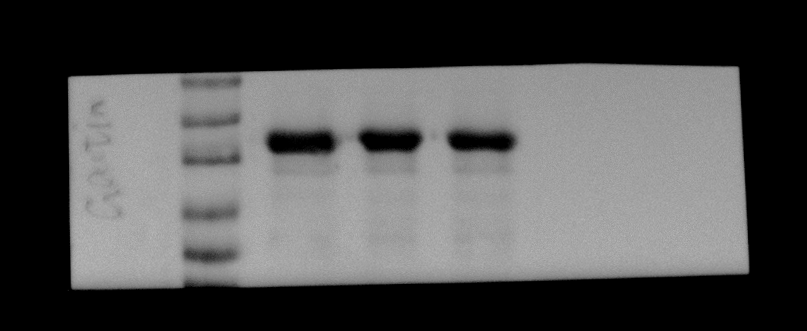

Supplement: Supplemental Information 5 [file peerj-12-18489-s005.zip › Uncropped Blots/figure7A/actin-1.tif]

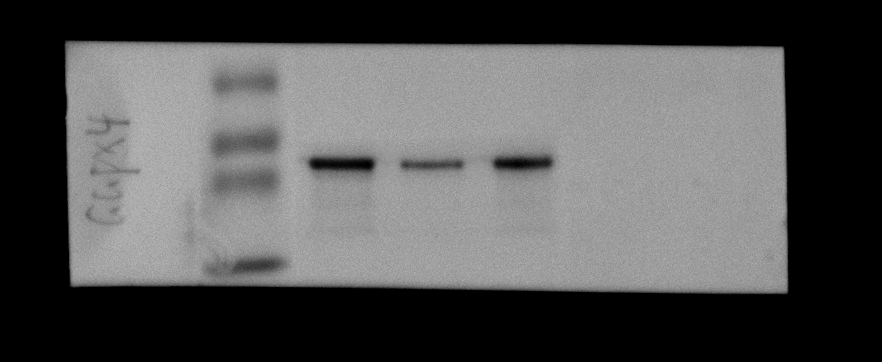

Supplement: Supplemental Information 5 [file peerj-12-18489-s005.zip › Uncropped Blots/figure8A/GPX4-2.tif]

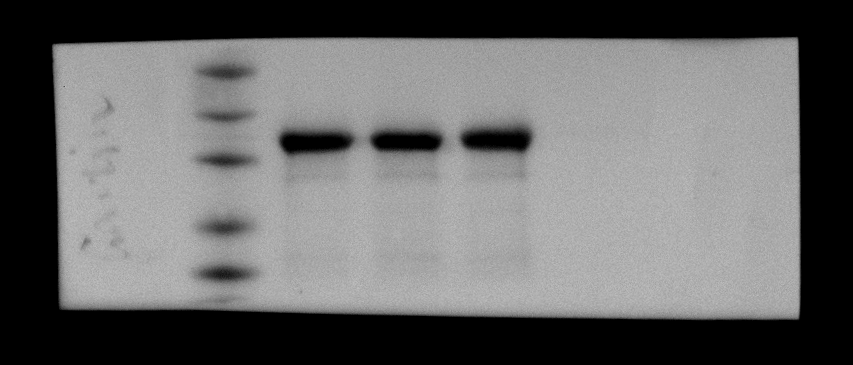

Supplement: Supplemental Information 5 [file peerj-12-18489-s005.zip › Uncropped Blots/figure8A/actin-2.tif]
